# Buckling and shape control of prestressable trusses using optimum number of actuators

**DOI:** 10.1038/s41598-023-30274-y

**Published:** 2023-03-07

**Authors:** Ahmed Manguri, Najmadeen Saeed, Marcin Szczepanski, Robert Jankowski

**Affiliations:** 1 Faculty of Civil and Environmental Engineering, Gdansk University of Technology, Gdańsk, Poland; 2 Civil Engineering Department, University of Raparin, Rania, Kurdistan Region Iraq; 3 Civil Engineering Department, Tishk International University, Erbil, Kurdistan Region Iraq

**Keywords:** Civil engineering, Mechanical engineering

## Abstract

This paper describes a method to control the nodal displacement of prestressable truss structures within the desired domains. At the same time, the stress in all members is unleashed to take any value between the allowable tensile stress and critical buckling stress. The shape and stresses are controlled by actuating the most active members. The technique considers the members’ initial crookedness, residual stresses, and slenderness ratio (S). Furthermore, the method is premeditated so that the members with an S between 200 and 300 can carry only tension before and after adjustment (i.e., the maximum compressive stress for the members with an S between 200 and 300 is zero). In addition, the derived equations are coupled with an optimization function that relies on five optimization algorithms (interior-point, trust-region-reflective, Sequential quadratic programming (SQP), SQP-legacy, and active-set). The algorithms identify and then exclude inactive actuators in the subsequent iterations. The technique is applied to several examples, and its results are compared with a quoted method in the literature.

## Introduction

Buckling is considered a severe issue in compression members; it should be carefully dealt with in structural design^1^, especially in trusses^2^. Deformed geometry and displacements also affect the performance of systems^3^. Correspondingly, buckling is crucial in designing pin-jointed assemblies and is even more vital during the reshaping process. Hitherto, researchers worked on reshaping and even stress controlling; however, they neglected the issue of buckling and/or slenderness of the members on the structure’s health^4–8^. The shape-reforming idea, which is the process of controlling deformation and shape restoration^9^, was introduced by Weeks^10^ and then analytically formulated by Haftka and Adelman^11^. The position of nodes is sensitive to the length bar changing^12,13^, which is done by actuators.

Furuya and Haftka^4^ proposed two simple algorithms to statically reshape a 150-bar space truss without regard to the status of stresses. The shape of various structures, for example, intelligent structures^6^ and trusses^7^, has been controlled while the stress in members is underestimated. Kwan and Pellegrino^14^ stated that actuating active bars, which are the key element of controlling the structures in terms of geometry and stress, may significantly affect stress redistribution in members. Kawaguchi et al.^5^ attempted to control the displacement and internal bar force of a 3-bar prestressable truss while undervalued buckling and members’ slenderness. Furthermore, buckling and slenderness effects were also disregarded in the study conducted by Saeed and Kwan^8^ to shape and stress control of indeterminate structures.

The researchers worked on truss optimization; they neglected the effects of initial crookedness, residual stresses, and slenderness. Size and/or topology optimization was performed for several numerical models, such as a 72-bar truss^15–19^. At the same time, most members were slender, and their compressive strength by far less than the induced stress for the loading cases. In addition, cross-sectional area optimization was performed for a 25-bar 3D truss^20–23^; most members' S was far greater than the allowable limit, and most compression members exceeded critical buckling stress. In this study, the members were designed based on their S, and their initial crookedness and residual stress were considered while reshaping structures.

Based on the aforementioned literature, it was concluded that the buckling problem was underestimated during the process of structures’ reshaping. Heretofore, in shape and stress control, it has been assumed that the compressive strength is the same amount as the tensile strength, and such cases are hardly achievable in practice. Therefore, in this paper, a method has been developed to control nodal displacements and strictly consider buckling in compression members simultaneously. In addition, the members with an S greater than 200 are not allowed to face compression, which is the aim of this research. Consequently, further improvement has been made using as few actuators as possible in coupling derived formulations with the optimization algorithms. Moreover, the further aim is to consider the residual stress and initial crookedness in members while displacement adjustment.

One of the main objectives of this work is to implement optimization algorithms. Optimization algorithms are performed to minimize resources^24–26^. For the sake of the economy and practice minimizing the number of actuators is a good point in the controlling process. Consequently, finding the optimum location for the actuators is highly recommended^14^. Researchers conducted studies to find the optimum number of actuators to control the configuration of a morphing wing scissor mechanism^27^, cable structures^28,29^, pin-jointed assemblies^30^, and spatial structures^31,32^. Meanwhile, the optimum location of redundant bars in trusses could be identified while they considered buckling effects, as stated by Jalihal et al.^33^.

Saeed and Kwan^8^ derived Eq. (1) to control shape and stress in prestressable structures simultaneously, but they neglected to consider limits for either joint displacements or axial bar forces. They also ignored the effect of buckling, which is a severe failure case in compression members. Using their technique, the user must input precise numbers for nodal displacements and bar axial forces for specific joints and members, respectively. Nonetheless, the considered structure should be monitored step by step in case any joints or members exceed their limits. Their method is time-consuming and could lead to mistakes made by the user. The mentioned problems in Saeed and Kwan^8^ work are solved in this study.

1 $$\left[ \begin{gathered} {\mathbf{Y}} \hfill \\ {\mathbf{Z}} \hfill \\ \end{gathered} \right]{\mathbf{e}}_{{\mathbf{o}}} = \left[ \begin{gathered} {\mathbf{d}} - {\mathbf{d}}_{{\mathbf{p}}} \hfill \\ {\mathbf{t}}_{{\mathbf{p}}} - {\mathbf{t}} \hfill \\ \end{gathered} \right]$$

${\mathbf{Y}}$ is the matrix that links the observed nodal joints and the members embedded with actuators while ${\mathbf{Z}}$ connects the prescribed axial forces in members and those embedded with actuators. ${\mathbf{e}}_{{\mathbf{o}}}$ is the amount of actuation needed to attain the targeted displacement (${\mathbf{d}}$) and axial force (${\mathbf{t}}$), while ${\mathbf{d}}_{{\mathbf{p}}}$ and ${\mathbf{t}}_{{\mathbf{p}}}$ are the induced nodal displacements and internal bar forces due to nodal loadings only.

The paper's outline is as follows: The study's introduction is presented in Section "Introduction". It is followed by formulating the current technique and applying it to an illustrative example; in addition, nodal displacement control and axial force control in members are also presented in Section "Method formulation and an illustrative example". Moreover, Section "Validation of the current technique" presents the verification of the current technique based on the studies discussed in the literature. While, in Section "Implementation of the current technique", the presented technique is applied on a 12-bar truss and a 15-bar truss. Finally, the drawn conclusion of the study summarized in Section "Conclusion".

## Method formulation and an illustrative example

**Figure 1:**
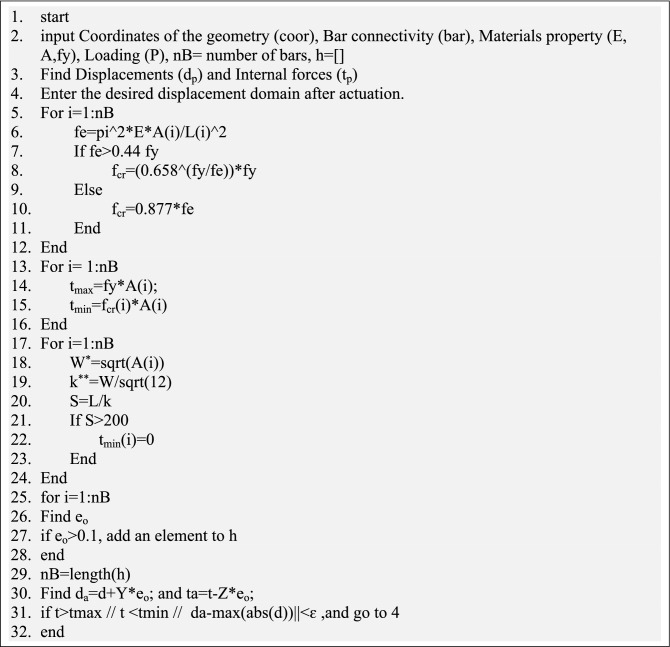
The pseudocode of the presented method.

**Figure 2:**
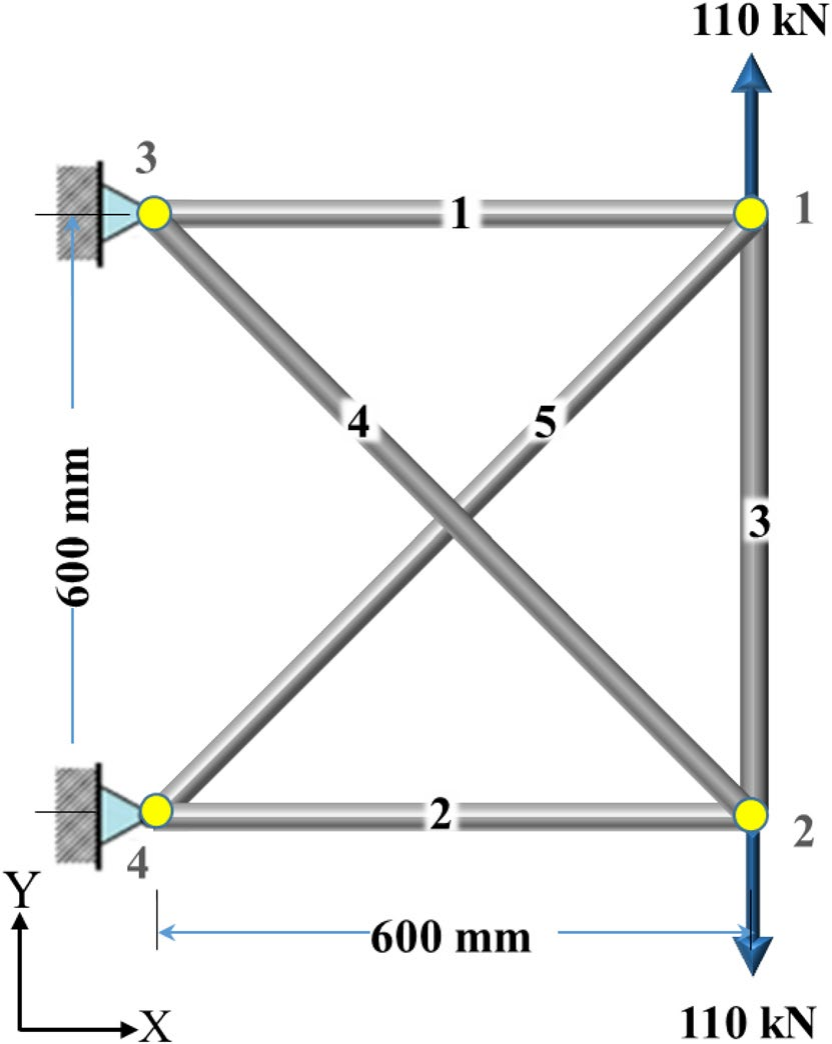
The 5-bar truss with one state of self-stress.

In this section, the new technique is derived, and a pseudocode (see Fig. 1) is presented to demonstrate the steps of the current technique. In addition, the equations are developed and applied to a 5-bar truss shown in Fig. 2. The numerical model has only one degree of indeterminacy; its members have a modulus of elasticity of 70 GPa and square cross-sections of ${[13}\quad {13}\quad {20}\quad {10}\quad {10]}\;{\text{mm}}$. The dimensions were selected based on the yield stress (fy = 276 MPa) and slenderness ratio (S), in which the compression members are limited to having an S of up to 200, while the tension members can have an S of up to 300^34^.

### Nodal displacement control

The displacement part of Eq. (1) can be expressed as

2 $${\mathbf{Y}}_{{\mathbf{1}}} {\mathbf{e}}_{o} {\mathbf{ + d}}_{{\mathbf{p}}} {\mathbf{ = d}}$$

${\mathbf{Y}}_{{\mathbf{1}}} = active({\mathbf{Y}})$, ${\mathbf{Y}}_{{\mathbf{1}}}$ is found by the optimization algorithm by identifying the active members that significantly influence the prescribed nodal joints. The sum of the absolute values of Column 3 in matrix **Y** is less than that of the other columns; thus, it can be said that Member 3 is the most effective member to change the position of nodes 1 and 2.

$$\begin{gathered} \quad \quad \quad \quad \quad \quad {\text{Embeded members with actuators}} \hfill \\ \quad \quad \quad \quad \quad \quad 1\quad \quad \quad \quad \quad 2\quad \quad \quad \quad 3\quad \quad \quad \quad 4\quad \quad \quad \quad \quad 5 \hfill \\ {\mathbf{Y}}_{{\mathbf{1}}} = \left[ \begin{gathered} x_{1} \hfill \\ y_{1} \hfill \\ x_{2} \hfill \\ y_{2} \hfill \\ \end{gathered} \right]\left[ {\begin{array}{*{20}l} {{\text{0}}{\text{.91655}}} \hfill & { - {\text{0}}{\text{.08345}}} \hfill & { - {\text{0}}{\text{.08345}}} \hfill & {{\text{0}}{\text{.11802}}} \hfill & {{\text{0}}{\text{.11802}}} \hfill \\ { - {\text{0}}{\text{.51763}}} \hfill & {{\text{0}}{\text{.48237}}} \hfill & {{\text{0}}{\text{.48237}}} \hfill & { - {\text{0}}{\text{.68217}}} \hfill & {{\text{0}}{\text{.73204}}} \hfill \\ { - {\text{0}}{\text{.083454}}} \hfill & {{\text{0}}{\text{.91655}}} \hfill & { - {\text{0}}{\text{.08345}}} \hfill & {{\text{0}}{\text{.11802}}} \hfill & {{\text{0}}{\text{.11802}}} \hfill \\ { - {\text{0}}{\text{.48237}}} \hfill & {{\text{0}}{\text{.51763}}} \hfill & { - {\text{0}}{\text{.48237}}} \hfill & { - {\text{0}}{\text{.73204}}} \hfill & {{\text{0}}{\text{.68217}}} \hfill \\ \end{array} } \right] \hfill \\ \end{gathered}$$

Due to the loadings shown in Fig. 2, the transposed vector of the induced nodal displacements becomes ${\mathbf{d}}_{{\mathbf{p}}} ^{\prime} = \left[ { - \,{0}{\text{.196}}\quad {1}{\text{.137}}\quad - \,{0}{\text{.196}}\quad - \,{1}{\text{.137}}} \right]\;{\text{mm}}$. Firstly, all free nodes are unleashed to take a value within a given limit ([− 0.5 to 0.5] mm); thus, Eq. (2) becomes

3 $$- \,{\mathbf{d}} \le {\mathbf{Y}}_{{\mathbf{1}}} {\mathbf{e}}_{o} {\mathbf{ + d}}_{{\mathbf{p}}} \le {\mathbf{d}}$$

For the given example, the upper limit is ${\mathbf{d}}^{\prime} = \left[ {{0}{\text{.5}}\quad {0}{\text{.5}}\quad {0}{\text{.5}}\quad {0}{\text{.5}}} \right]\;{\text{mm}}$, and the lower limit is $- \,{\mathbf{d}}^{\prime} = \left[ { - \,{0}{\text{.5}}\quad - \,{0}{\text{.5}}\quad - \,{0}{\text{.5}}\quad - \,{0}{\text{.5}}} \right]\;{\text{mm}}$.

The inequality relationship in Eq. (3) can be solved as below, based on the fmincon function in MATLAB^35^.

4 $$c = \left[ {\begin{array}{*{20}c} {{\mathbf{Y}}_{{\mathbf{1}}} {\mathbf{e}}_{o} {\mathbf{ + dd}}_{{\mathbf{1}}} } \\ { - \,{\mathbf{Y}}_{{\mathbf{1}}} {\mathbf{e}}_{o} - \,{\mathbf{dd}}_{{\mathbf{2}}} } \\ \end{array} } \right]$$

In Eq. (4), the expression (c = []) is used to find e_o_ such that displacements after applying actuation remain in the required domain.

When ${\mathbf{dd}}_{{\mathbf{1}}} {\mathbf{ = [d}}_{{\mathbf{p}}} \; - \;{\mathbf{d]}}$ and ${\mathbf{dd}}_{{\mathbf{2}}} {\mathbf{ = [d}}_{{\mathbf{p}}} {\mathbf{ + d]}}$, thus ${\mathbf{dd}}_{{\mathbf{1}}} ^{\prime} = \left[ { - \;{0}{\text{.696}}\quad {0}{\text{.637}}\quad - \;{0}{\text{.696}}\quad - \;{1}{\text{.637}}} \right]\;{\text{mm}}$ and ${\mathbf{dd}}_{2} ^{\prime} = \left[ {0.304\quad {1}{\text{.637}}\quad {0}{\text{.304}}\quad - \;{0}{\text{.637}}} \right]\;{\text{mm}}$.

### Axial force control in members

The internal force part of Eq. (1) is expressed as:

5 $${\mathbf{Z}}_{{\mathbf{1}}} {\mathbf{e}}_{{\mathbf{0}}} {\mathbf{ = t}}_{{\mathbf{p}}} - {\mathbf{t}}$$

In which ${\mathbf{Z}}_{{\mathbf{1}}} = active({\mathbf{Z}})$, ${\mathbf{Z}}_{{\mathbf{1}}}$ is found by the optimization algorithm via detecting the most active members that have a substantial effect on the targeted members. The minimum sum value of columns in **Z** matrix indicates the effectiveness of the member. Columns 1–3 have the smallest sum, but Member 3 was the most active in displacement control. Considering displacement and internal force simultaneously, Member 3 is selected as the most active member.

$$\begin{gathered} \quad \quad \quad \quad \quad \quad {\text{Embeded members with actuators}} \hfill \\ \quad \quad \quad \quad \quad 1\quad \quad \quad 2\quad \quad \quad \quad 3\quad \quad \quad 4\quad \quad \quad \quad 5 \hfill \\ {\mathbf{Z}}_{{\mathbf{1}}} = \left[ \begin{gathered} 1 \hfill \\ 2 \hfill \\ 3 \hfill \\ 4 \hfill \\ 5 \hfill \\ \end{gathered} \right]\left[ {\begin{array}{*{20}l} {{\text{1645}}} \hfill & {{\text{1645}}} \hfill & {{\text{1645}}} \hfill & { - \;{\text{2327}}} \hfill & { - \;{\text{2327}}} \hfill \\ {{\text{1645}}} \hfill & {{\text{1645}}} \hfill & {{\text{1645}}} \hfill & { - \;{\text{2327}}} \hfill & { - \;{\text{2327}}} \hfill \\ {{\text{1645}}} \hfill & {{\text{1645}}} \hfill & {{\text{1645}}} \hfill & { - \;{\text{2327}}} \hfill & { - \;{\text{2327}}} \hfill \\ { - \;{\text{2327}}} \hfill & { - \;{\text{2327}}} \hfill & { - \;{\text{2327}}} \hfill & {{\text{3290}}} \hfill & {{\text{3290}}} \hfill \\ { - \;{\text{2327}}} \hfill & { - \;{\text{2327}}} \hfill & { - \;{\text{2327}}} \hfill & {{\text{3290}}} \hfill & {{\text{3290}}} \hfill \\ \end{array} } \right] \hfill \\ \end{gathered}$$

The transposed vector of the induced axial forces due to external loadings shown in Fig. 2 becomes

${\mathbf{t}}_{{\mathbf{p}}} ^{\prime} = \left[ { - 3879\quad - 3879\quad 106120\quad 5485\quad 5485} \right]\;{\text{N}}$. Equation (5) can be expressed as

6 $${\mathbf{t = t}}_{{\mathbf{p}}} - {\mathbf{Z}}_{{\mathbf{1}}} {\mathbf{e}}_{o}$$

Equation (11) should be expressed as an inequality to allow the user to input the upper limit in tension and lower limit in compression, which varies based on the members’ S.

7 $${\mathbf{t}} \ge {\mathbf{t}}_{{\mathbf{p}}} - {\mathbf{Z}}_{{\mathbf{1}}} {\mathbf{e}}_{o} \ge - {\mathbf{t}}$$

The effectiveness of the current technique is that the allowable tensile and compressive forces are determined for each member, and for the allowable compressive force, initial crookedness, and residual stress of the members are considered. The detail of determining the limits are discussed in the following subsections.

#### Tension control

$${\mathbf{t}} = {\text{allowable tensile force (tten)}} = fy*A = \left[ {{46644}\quad {46644}\quad {11}0{4}00\quad {276}00\quad {276}00} \right]^{\prime}\;{\text{N}}$$

#### Buckling control (compression)

$- {\mathbf{t}} = {\text{ allowable compressive force (tcomp)}}$ and $tcomp = fcr*A$. There are two cases for determining critical buckling stress.

A. If $$\frac{L}{k} \le 4.71\sqrt{\frac{E}{Fy}} orFe \ge 0.44Fy$$.

$fcr = (0.658^{Fy/Fe} )Fy$ where inelastic buckling dominates the members’ behavior because of the presence of residual stresses in the member.

Where $Fe$ is the Euler load, $Fe = \frac{{\pi^{2} EA}}{{\left( L \right)^{2} }}$. The effective length coefficient is unity since the joints have a pin-pin connection.

B. if $$\frac{L}{k} > 4.71\sqrt{\frac{E}{Fy}} orFe < 0.44Fy$$.

$fcr = 0.877Fe$ is dominant in the case of long and slender members; thus, 0.877 account for initial crookedness.

Part B is the case of the members in this example. For the current example, ${\text{Fe}} = \left[ {{27}.0{27}\quad {27}.0{27}\quad {63}.{97}\quad {7}.{9962}\quad {7}.{9962}} \right]\;{\text{MPa}}$. Thus ${\text{fcr}} = \left[ { - {23}.{7}0{3}\quad - {23}.{7}0{3}\quad - {56}.{1}0{1}\quad - {7}.0{127}\quad - {7}.0{127}} \right]\;{\text{MPa}}$. Consequently, tcomp' = $\left[ { - {4}00{5}.{8}\quad - {4}00{5}.{8}\quad - {22441}\quad - {7}0{1}.{27}\quad - {7}0{1}.{27}} \right]\;{\text{N}}$.

However, in this example, none of the tension members change their phase to compression after shape restoration. It should be noted that the members with S > 200 should not carry a compression load. This condition should be strictly considered during the design process and post-adjustment. In the sense of stress control during shape restoration, another condition should be added: the allowable compression force for members with S greater than 200 is 0 N. In other words, members with S > 200 remain in tension or zero force after actuation. This phenomenon is experienced in the subsequent examples.

To minimize ${\mathbf{e}}_{o}$ based on the fmincon optimization technique, Eq. (7) is expressed as follows

8 $$c = \left[ {\begin{array}{*{20}c} { - {\mathbf{Z}}_{{\mathbf{1}}} {\mathbf{e}}_{o} {\mathbf{ + tt}}_{{\mathbf{1}}} } \\ {{\mathbf{Z}}_{{\mathbf{1}}} {\mathbf{e}}_{o} - {\mathbf{tt}}_{{\mathbf{2}}} } \\ \end{array} } \right]$$

Similar to Eq. (4), to keep the axial force within the domain after actuation of members, the inequality relationship in Eq. (7) is inserted to c = []. When ${\mathbf{tt}}_{{\mathbf{1}}} {\mathbf{ = [}} - \;{\mathbf{t}}_{{\mathbf{p}}} - {\mathbf{t]}}$ and ${\mathbf{tt}}_{{\mathbf{2}}} {\mathbf{ = [t}}_{{\mathbf{p}}} {\mathbf{ + t]}}$, in this case, ${\mathbf{tt}}_{{\mathbf{1}}} ^{\prime} = \left[ { - \;{127}\quad - \;{127}\quad {128561}\quad - \;{6186}\quad - \;{6186}} \right]\;{\text{N}}$ and ${\mathbf{tt}}_{{\mathbf{2}}} ^{\prime} = \left[ {{42766}\quad {42766}\quad {216520}\quad {33085}\quad {33085}} \right]\;{\text{N}}$.

Combining Eqs. (4 and 8) becomes

9 $$c = \left[ {\begin{array}{*{20}c} {{\mathbf{Y}}_{{\mathbf{1}}} {\mathbf{e}}_{o} {\mathbf{ + dd}}_{{\mathbf{1}}} } \\ { - {\mathbf{Y}}_{{\mathbf{1}}} {\mathbf{e}}_{o} - {\mathbf{dd}}_{{\mathbf{2}}} } \\ { - {\mathbf{Z}}_{{\mathbf{1}}} {\mathbf{e}}_{o} {\mathbf{ + tt}}_{{\mathbf{1}}} } \\ {{\mathbf{Z}}_{{\mathbf{1}}} {\mathbf{e}}_{o} - {\mathbf{tt}}_{{\mathbf{2}}} } \\ \end{array} } \right]$$

In this study, the fmincon function tries to find eo such that the displacements and axial forces remain in the required domains after prestressing. The relationship in Eq. (9) is subjected to Eq. (10), while Eq. (11) is well-preserved.

10 $$\min f(x) = \sum\limits_{i = 1}^{n} {e_{0i} }$$

where $n$ is the number of actuators. Equation (10) is the fmincon function^35^ that relies on five optimization algorithms (interior-point, trust-region-reflective, Sequential quadratic programming (SQP), SQP-legacy, and active-set). The fmincon function minimizes the number of actuators by identifying inactive members and excluding them in the subsequent iterations. In this study, the actuators with less than 0.1 mm actuation are assumed to be passive as illustrated in Fig. 1.

11 $$L_{b} \le {\mathbf{e}}_{0} \le U_{b}$$

$L_{b}$ and $U_{b}$ are lower and upper actuation limits. In this example, the actuators were allowed to have actuation within − 5 to 5 mm. $L_{b}$ and $U_{b}$ were assigned based on the ability of the actuators and the length of the members $- 5 \le {\mathbf{e}}_{o} \le 5$.

For the first iteration, all members are assumed to participate as actuators for controlling the nodal displacements and axial forces in members. The inputs were given to a code prepared in MATLAB; then, the total ${\mathbf{e}}_{{\mathbf{o}}}$ was found to be 1.3207 mm. ${\mathbf{e}}_{{\mathbf{o}}} ^{\prime} = \left[ {{2}{\text{.2148e}} - {07}\quad {2}{\text{.1738e}} - {07}\quad - {1}{\text{.3207}}\quad - {3}{\text{.1263e}} - {07}\quad - {3}{\text{.1325e}} - {07}} \right]\;{\text{mm}}$.

The result proves that Member 3 is the most active member; thus, the size of matrix Y1 and Z1 is (4 × 1) and (5 × 1), respectively. The algorithm excludes inactive members in the second iteration, and only Member 3 remains. Thus ${\mathbf{e}}_{{\mathbf{o}}} ^{\prime} = \left[ {0\quad {0}\quad - \,{1}{\text{.3206}}\quad {0}\quad {0}} \right]\;{\text{mm}}$ with a total ${\mathbf{e}}_{{\mathbf{o}}}$ of 1.3206 mm. The transposed nodal displacement vector after actuation (${\mathbf{d}}_{{\mathbf{a}}}$) can be found in the expression below.

12 $${\mathbf{d}}_{{\mathbf{a}}} {\mathbf{ = d}}_{{\mathbf{p}}} {\mathbf{ + Ye}}_{{\mathbf{o}}}$$

In this case, ${\mathbf{d}}_{{\mathbf{a}}} ^{\prime} = \left[ { - \,{0}{\text{.09}}\quad {0}{\text{.50}}\quad - \,{0}{\text{.09}}\quad - \,{0}{\text{.50}}} \right]\;{\text{mm}}$. It can be seen that none of the nodes exceed the limit [− 0.5 + 0.5] mm; the actuation of members is also within the domain. The members’ axial force after actuation (${\mathbf{t}}_{{\mathbf{a}}}$) is determined as follows

13 $${\mathbf{t}}_{{\mathbf{a}}} {\mathbf{ = t}}_{{\mathbf{p}}} - {\mathbf{Ze}}_{{\mathbf{o}}}$$

**Figure 3:**
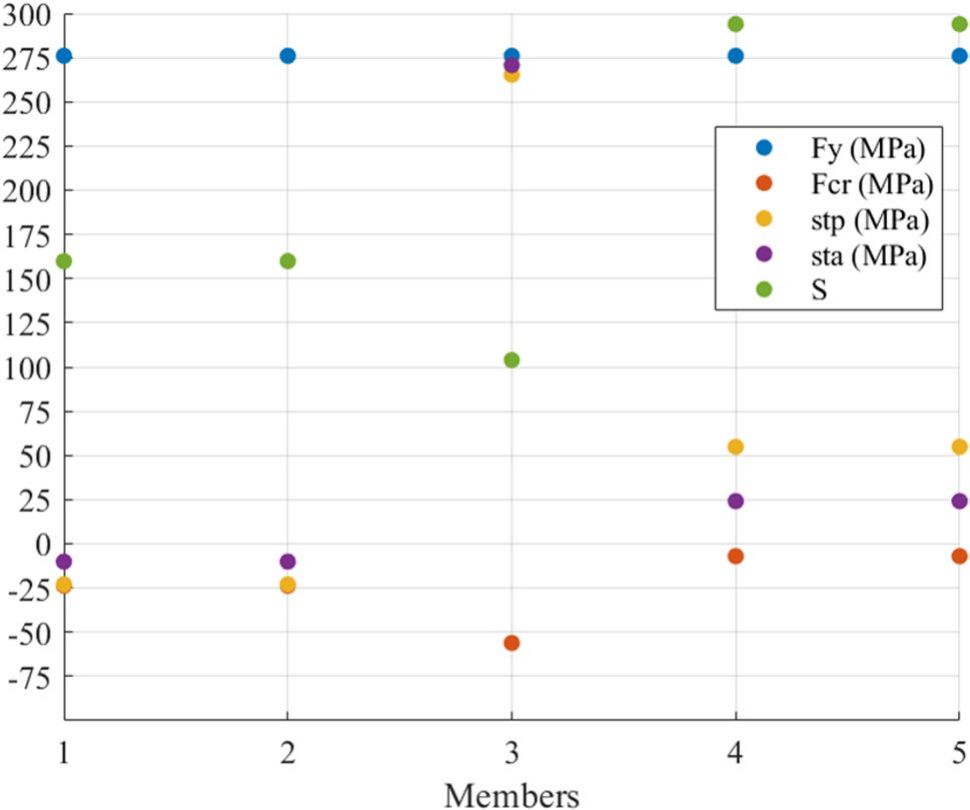
The stresses and S of the members of the 5-bar truss before and after actuation.

In this example, ${\mathbf{t}}_{{\mathbf{a}}} ^{\prime} = \left[ { - \,{1705}\quad - \,{1705}\quad {108290}\quad {2412}\quad {2412}} \right]\;{\text{N}}$. Figure 3 shows the members’ stresses and the S to see the situation of axial stresses before (${\mathbf{St}}_{{\mathbf{p}}}$) and after actuation (${\mathbf{St}}_{{\mathbf{a}}}$) and compare them with the yield and critical compressive stresses. It can be seen that ${\mathbf{St}}_{{\mathbf{p}}}$ and ${\mathbf{St}}_{{\mathbf{a}}}$ are within the allowed domain; furthermore, the members with S greater than 200 did not face compression. In terms of Member 3, it has an S of around 100 while the member is in tension. Its cross-sectional area was selected based on the induced tensile stress which is close to the materials yield stress. Numerically speaking, the tensile stress in Members 4 and 5 changed from almost 55 MPa to around 25 MPa, and the compressive stress in Members 1 and 2 changed from 23 to 10 MPa while their compressive strength is 25 MPa.

## Validation of the current technique

**Figure 4:**
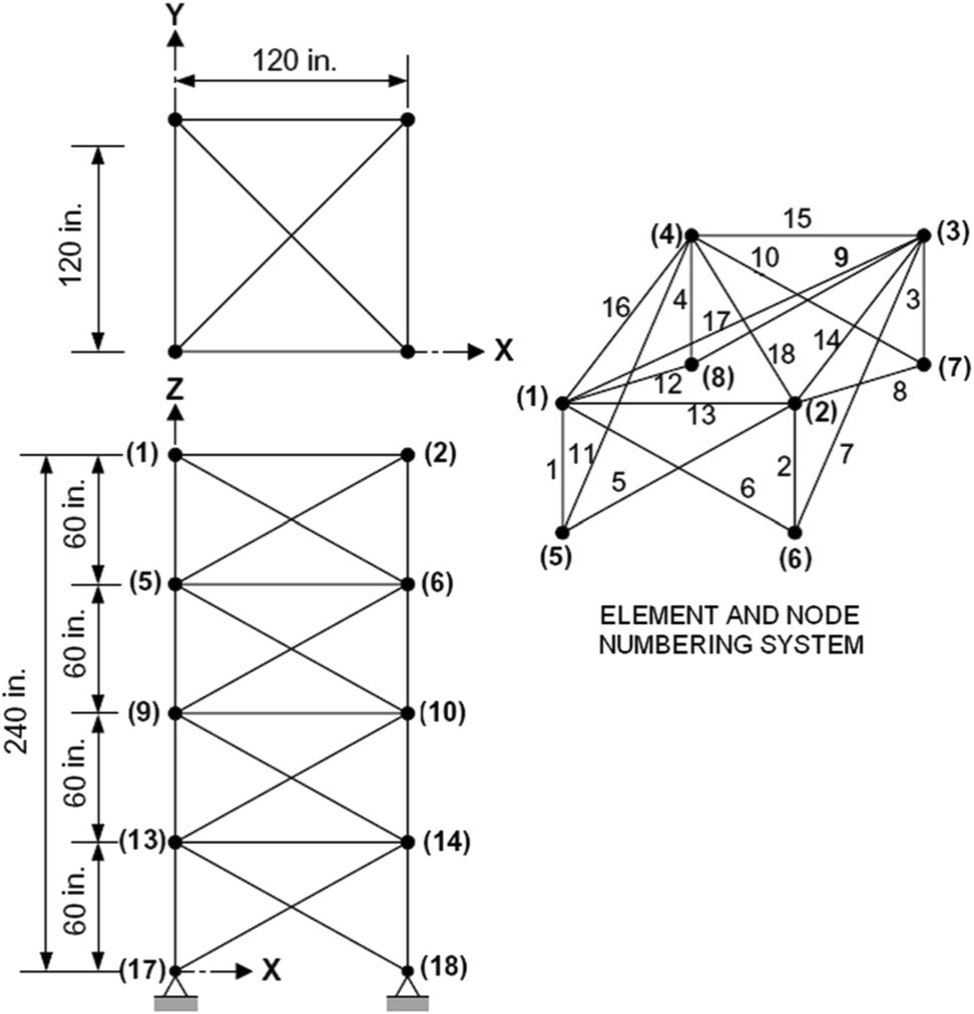
The 72-bar 3D truss^20^.

The current technique was applied to a previously studied example by Farshi and Alinia-Ziazi^20^. They neglected the issues of buckling and slenderness ratio in their study. Most of the members would fail due to buckling for the given loading. In addition, according to the standards and even in practice, S for compression and tension members must not exceed 200 and 300, respectively. The domain of the nodal displacement can be preserved using the current technique. Meanwhile, none of the members face buckling, as presented in this example. A 72-bar 3D aluminum truss shown in Fig. 4 with an elasticity modulus of 70,000 MPa was loaded from the top joints in the Z-direction with 22241N downward. The numerical example was examined to optimize the size of the members by several researchers, including Lee and Geem^36^, and Sedaghati^15^, but they neglected to consider buckling effects and slenderness ratio limits. With the selected cross-sectional area and the loading case, all compression members would fail due to buckling; nonetheless, the S of all members is far greater than the allowable limits. In this study, the members' areas (see Table 1) were selected based on the axial stresses and slenderness ratio limits. It was noted that the tension members could have an S of up to 300, while the S of compression members cannot exceed 200.

**Table 1:** The designed area for the 72-bar truss.

Members	Area (mm^2^)	Members	Area (mm^2^)
1–4	696.7	37–40	696.7
5–12	3483.9	41–48	3483.9
13–16	1238.7	49–52	1238.7
17–18	2477.4	53–54	2477.4
19–22	1254.2	55–58	696.7
23–30	1548.4	59–66	3483.9
31–34	1238.7	67–70	1238.7
35–36	2477.4	71–72	2477.4

Table 2 shows the nodal displacements of joints in the direction of loading pre (d_p_) and post (d_a_)-actuation, the movements of the other directions were insignificant. It can be seen from the table, the maximum displacement (− 1.83 mm) occurred in the Z-direction of the top four joints. The goal was to adjust the displacements to up to ± 0.5 mm movement meanwhile keeping the stress in all members within the yield stress, and critical buckling stress. The targets were attained in four iterations by actuating only 45 active members $\left[ {{2}\quad {4} - {9}\quad {11} - {14}\quad {19}\quad {21}\quad {24} - {27}\quad {29} - {31}\quad {37}\quad {39} - {42}\quad {44} - {48}\quad {52} - {54}\quad {58} - {62}\quad {64}\quad {66} - {7}0\quad {72}} \right]$ with total actuation of 25.6 mm. One can see that from Table 2, the displacement constraints were preserved after applying actuation, in other words, d_a_ has not exceeded the limit, which is ± 0.5 mm.

**Table 2:** Nodal displacement of Z-direction before and after actuation.

Joints	d_p_ (mm)	d_a_ (mm)	Joints	d_p_ (mm)	d_a_ (mm)
1	− 1.83	0	9	− 0.99	0.3
2	− 1.83	0.08	10	− 0.99	0.36
3	− 1.83	0.07	11	− 0.99	0.18
4	− 1.83	− 0.3	12	− 0.99	0.22
5	− 1.35	− 0.21	13	− 0.47	0.19
6	− 1.35	− 0.17	14	− 0.47	0.23
7	− 1.35	− 0.14	15	− 0.47	0.2
8	− 1.35	− 0.31	16	− 0.47	0.09

Table 3 shows the comparison between the current technique and the quoted method. The table shows the yield stress and allowable compressive stress as well as the allowable S based on the members’ stress states and lengths. Column 6, which represents Farshi and Alinia-Ziazi^20^ study results, shows that all compression members exceed the limit; meanwhile, Column 8 shows that sixty-four members’ S is greater than the allowable. On the other hand, Columns 13 and 15 of Table 3 present the current methods’ results. The results confirm that neither stress nor S passed the limits using the current method.

**Table 3:** Comparison between previous studies and the presented method.

			Farshi and Alinia-Ziazi^20^					Current study					
1	2	3	4	5	6	7	8	10	11	12	13	14	15
Members	Fy (MPa)	Allowable S	Stp (MPa)	Fcr (MPa)	C4/C2 or C4/C5	S	C7/C3	Stp (MPa)	Sta (MPa)	Fcr (MPa)	C11/C12 or C11/C2	S	C14/C3
1–4	172	200	− 172.3	− 2.16	79.71	525	2.6	− 21.8	9.5	− 14.9	− 0.64	200	1
5–12	172	200	− 15.3	− 1.51	10.20	629	3.2	− 2.3	− 9.3	− 14.9	0.62	200	1
13–16	172	300	9.2	− 1.42	0.05	648	2.2	2.4	9.9	0	0.06	300	1
17–18	172	300	9.2	− 0.98	0.05	779	2.6	2.4	9.4	0	0.05	300	1
19–22	172	200	− 67.6	− 7.23	9.34	287	1.4	− 16.5	− 24.6	− 26.8	0.91	149	0.7
23–30	172	300	2.0	− 1.43	0.01	646	2.2	− 1.1	6.2	0	0.04	300	1
31–34	172	300	38.5	− 0.35	0.22	1315	4.4	2.9	5.3	0	0.03	300	1
35–36	172	300	38.5	− 0.17	0.22	1859	6.2	2.9	7.8	− 14.9	0.05	300	1
37–40	172	200	− 26.4	− 17.52	1.51	185	0.9	− 23.5	4.5	− 14.9	0.03	200	1
41–48	172	200	− 2.2	− 1.41	1.52	650	3.3	− 1.9	− 8.1	− 14.9	0.55	200	1
49–52	172	300	0.3	− 0.35	0.00	1315	4.4	2.5	6.5	0	0.04	300	1
53–54	172	300	0.3	− 0.17	0.00	1859	6.2	2.5	5.0	0	0.03	300	1
55–58	172	200	− 18.1	− 26.06	0.70	151	0.8	− 21.1	9.1	− 14.9	0.05	200	1
59–66	172	200	− 0.6	− 1.42	0.45	649	3.6	− 2.4	− 9.2	− 14.9	0.61	200	1
67–70	172	300	7.5	− 0.35	0.04	1315	4.4	4.5	21.2	0	0.12	300	1
71–72	172	300	7.5	− 0.17	0.04	1859	6.2	4.5	15.7	0	0.09	300	1

Numerically speaking, the stress in Members 1–4 is − 172.3 MPa, while the allowable compressive stress is 2.16 MPa. The S in these members is 2.6 times greater than the acceptable value. Similarly, the stress in Members 5–12 and 19–22 is about ten times greater than the compressive strength. Using the current method, all members are safe regarding stress and slenderness. The stress in Members 23–30 is compression, while the members' slenderness ratio is 300. After applying actuation, the members’ stress changed to tension since the method guarantees that members with S > 200 do not carry compression.

**Figure 5:**
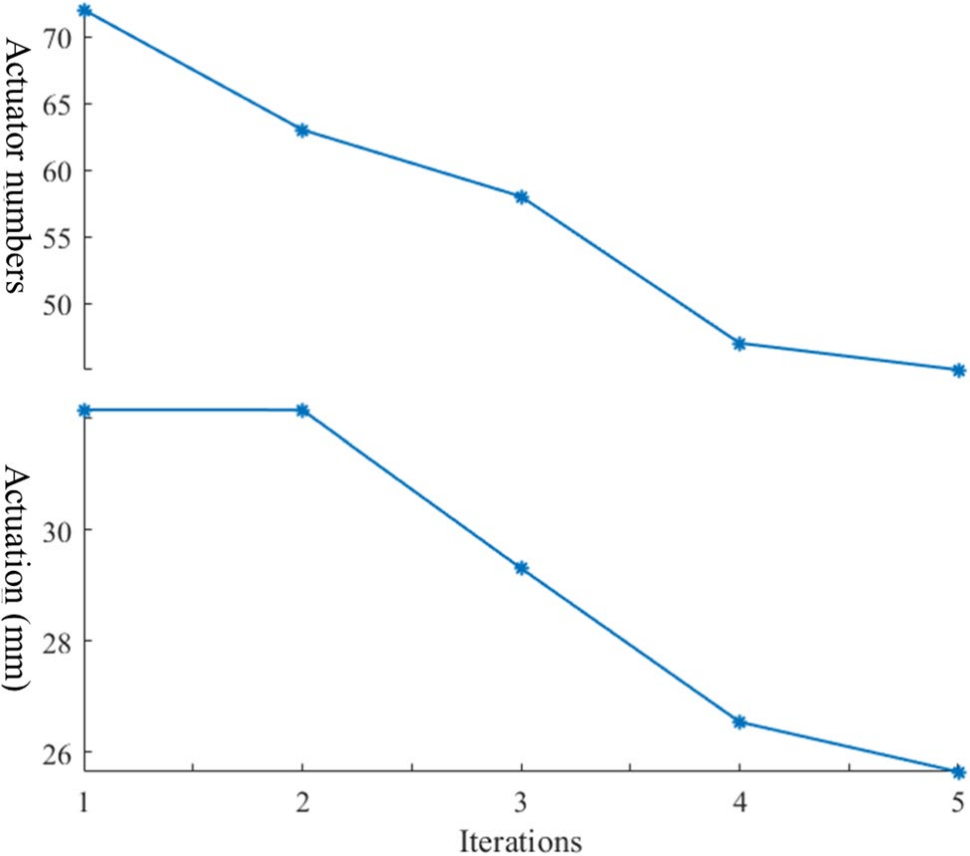
Optimization of the number of actuators and actuation for the 72-bar truss.

The algorithm started with using all members as actuators; then, in the several iterations, the inactive members were excluded meanwhile the targets were unchanged. For the 72-bar truss, the minimum possible actuator number was 45, with the optimum amount of actuation of 25.6 mm, as presented in Fig. 5. The figure illustrates that the number of active actuators dropped almost with the same slope between the first and third iterations. Whereas there was a sharp and negligible decrease in the number of active actuators between the third to four and fourth to fifth iterations, respectively. In terms of changes in the total actuation in the five iterations, the difference was negligible between the first two. While the total actuation steeply and linearly declined from the second to the fourth iteration. Moreover, there was a slight decrease in the total actuation between the fourth and the last iterations.

## Implementation of the current technique

In order to present the adequacy of the current technique, it is applied to two trusses with different geometries.

### *Example 1*

**Figure 6:**
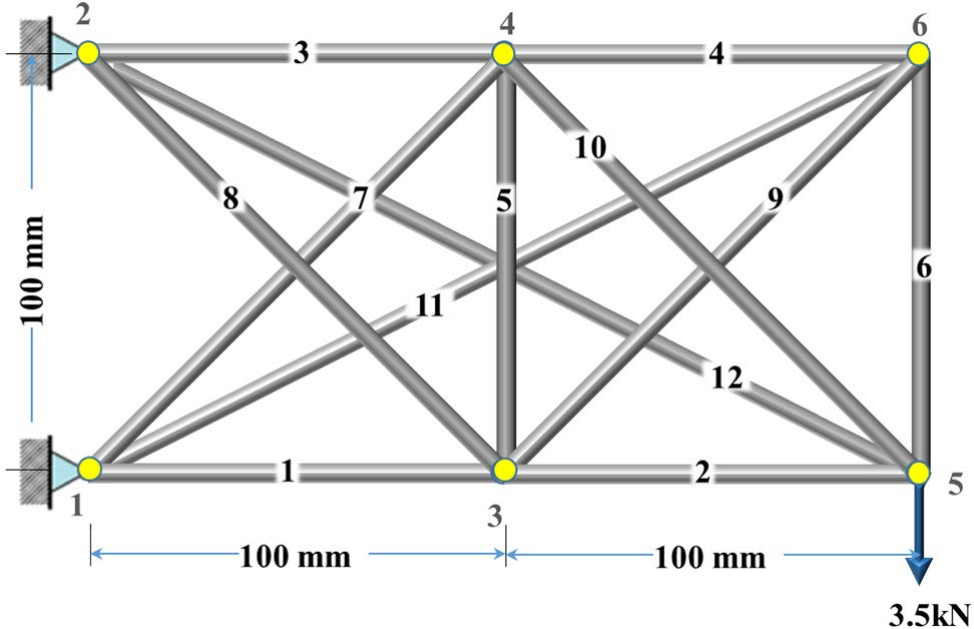
The 12-bar aluminum truss with four states of self-stress.

A 12-bar aluminum truss shown in Fig. 6 with four states of self-stress was designed based on the tensile and compressive stresses as well as S. The members 1–12 have E = 70,000 MPa and ${\text{Area}} = \left[ {{1}.{47}\quad {23}.0{4}\quad {17}.{64}\quad {9}.0\quad {9}.0\quad {9}.0\quad {28}.{19}\quad {9}.0\quad {24}.{5}0\quad {9}.0\quad {35}.{28}\quad {9}.0} \right]\;{\text{mm}}^{{2}}$ , respectively. The maximum displacement occurred at Joint 5y, which was 2.01 mm, as presented in Table 4. It was planned to adjust the displacements to up to ± 0.5 mm in all joints and keep the tensile stress within the yield stress and the compressive stress below the critical buckling stress. It should be noted that the members with S greater than 200 cannot carry a compression force either before or after adjustment. Table 4 shows that the goals were achieved in terms of nodal displacements. Numerically speaking, the displacement of joints 3–6 in the y direction before adjustment was $\left[ { - 0.{6}\quad - 0.{59}\quad - {2}.0{1}\quad - {1}.{93}} \right]\;{\text{mm}}$, respectively. After actuating Members 1, 3, and 11 with $\left[ {0.{54}\quad - 0.{47}\quad 0.{24}} \right]\;{\text{mm}}$ respectively, the nodal displacements of joints 3–6 in y direction become $\left[ { - 0.{14}\quad - 0.{13}\quad - 0.{5}\quad - 0.{24}} \right]\;{\text{mm}}$, respectively. To bring up the joints 5 and 6, Members 1 and 11 were lengthened, and Member 3 was shortened, while the amount of axial force in all members was considered to stay within the allowable limit.

**Table 4:** Nodal displacements of the 12-bar truss pre and post-adjustment.

Joints	Directions	${\mathbf{d}}_{{\mathbf{p}}}$(mm)	${\mathbf{d}}_{{\mathbf{a}}}$(mm)
3	x	− 0.20	0.34
	y	− 0.60	− 0.14
4	x	0.39	− 0.07
	y	− 0.59	− 0.13
5	x	− 0.37	0.19
	y	− 2.01	− 0.50
6	x	0.72	0.27
	y	− 1.93	− 0.24

**Figure 7:**
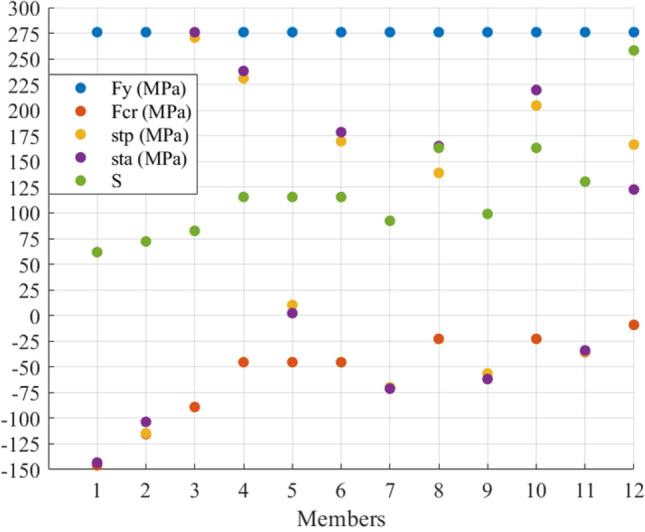
Stress redistribution and S of members of the 12-bar truss.

Regarding the stresses, Fig. 7 shows the members’ stress states, limits and S before and after actuation. The figure illustrates that all members stayed within their limits with slight changes in the stresses in Members 8, 10, and 12. One can see that, the tensile stress in Member 3 has increased since the member has been shortened. However, Members 1 and 11 are supposed to increase their compressive stress due to their lengthening; their axial stress remains under the allowable compressive stress. Furthermore, a noticeable increase in the tensile stress in Members 3, 4, 6, and 10 can be seen after applying e_o_ due to shortening Member 3 and lengthening Members 1 and 11.

### *Example 2*

**Figure 8:**
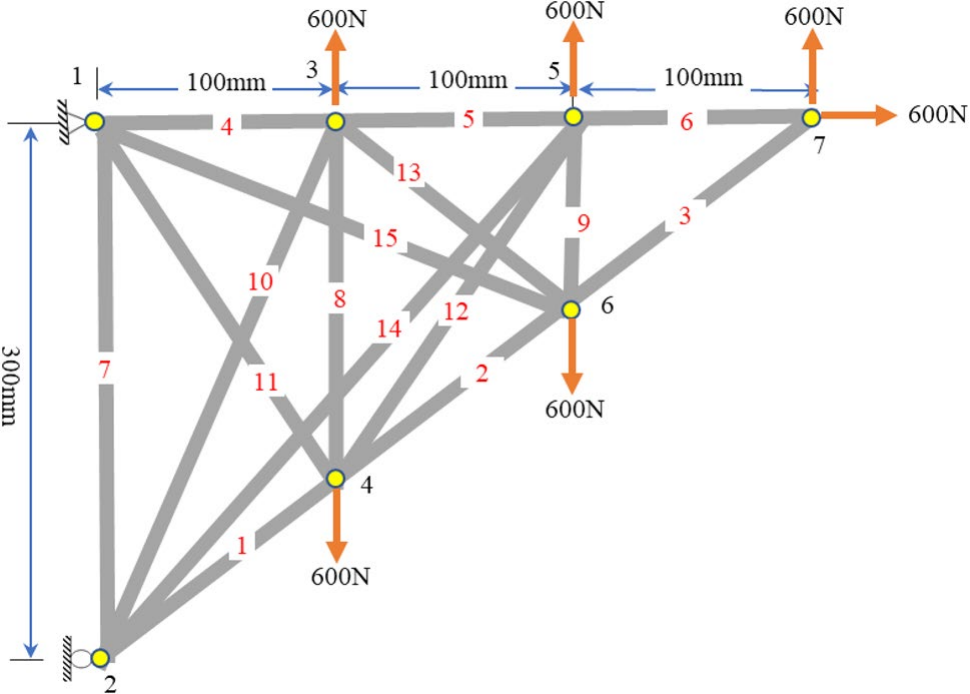
The 15-bar steel truss with four self-stress states.

**Figure 9:**
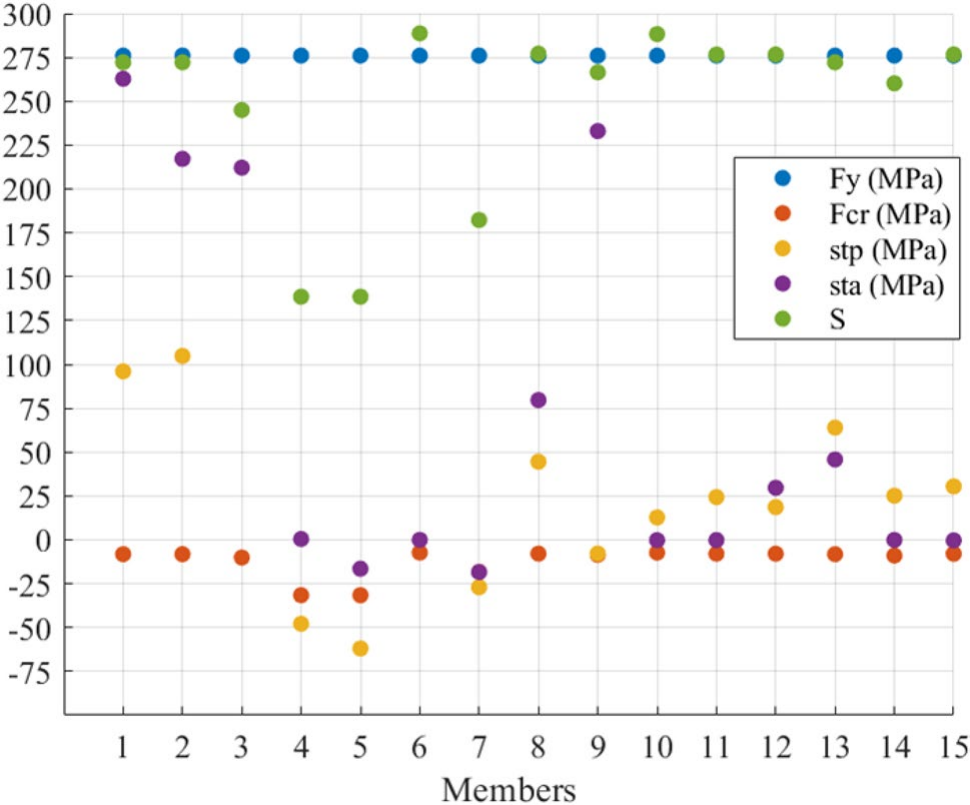
Stress distribution of the 15-bar truss members.

A 15-bar steel truss shown in Fig. 8 with E = 200,000 MPa was loaded at all free joints. In this example, if the established loads were applied to the structure before actuation, some members would fail, as illustrated in Fig. 9. If the loads were applied before the actuation, Members 4, 5, and 7 would experience the axial stresses − 48, − 62, − 27 MPa respectively, while their compressive strengths are − 32, − 32 and − 18 MPa respectively. By applying the current technique by actuating members 1, 2, 8, 10, and 13 by $\left[ { - 0.{7}0\quad - 0.{35}\quad - 0.{29}\quad - 0.{22}\quad - 0.{24}} \right]\;{\text{mm}}$, respectively, the axial stress in Members 4, 5, and 7 become 0, − 30, and − 18 MPa, respectively. In addition, Member 9 with S = 266 would experience compressive stress of − 8 MPa, before actuation, but using the current technique, the member remains in tension with 233 MPa. Moreover, Members 10, 11, 14, and 15 have S greater than 200; their axial stress after actuation is almost zero since they cannot carry compression. The figure shows that all members are within the limit of corresponding Fcr, and Fy after actuation.

In terms of nodal displacements, the joints were unleashed to take any position within ± 0.5 mm displacement. Table 5 shows the nodal displacements of the free joints in the X- and Y directions due to external loading only (d_p_) and due to loading and member actuation (d_a_). It can be seen that the maximum displacement occurred in the y-direction of Joint 7. The goal was to keep the nodal displacements in all joints in X and Y directions within ± 0.5 mm. Table 5 shows that the displacement targets were exactly attained after applying e_o_. Members 1 and 2 were shortened to reduce the y-displacement of joint 7 from 1.45 mm to 0.5 mm. Meanwhile, Members 8, 10, and 13 were shortened to adjust the axial stress of members 4, 5, and 7 from − 47 MPa to 0.35 MPa, − 61.9 MPa to − 29.7 MPa, and − 27.0 MPa to − 18.39 MPa respectively.

**Table 5:** Nodal displacements of the 15-bar free joints.

Joints	Directions	d_p_ (mm)	d_a_ (mm)	Joints	Directions	d_p_ (mm)	d_a_ (mm)
2	x	–	–	5	x	− 0.16	− 0.04
	y	0.12	0.08		y	0.38	0.11
3	x	− 0.07	0.00	6	x	0.30	− 0.04
	y	0.20	− 0.15		y	0.39	− 0.09
4	x	0.32	− 0.11	7	x	− 0.16	− 0.04
	y	0.07	− 0.05		y	1.45	0.50

**Figure 10:**
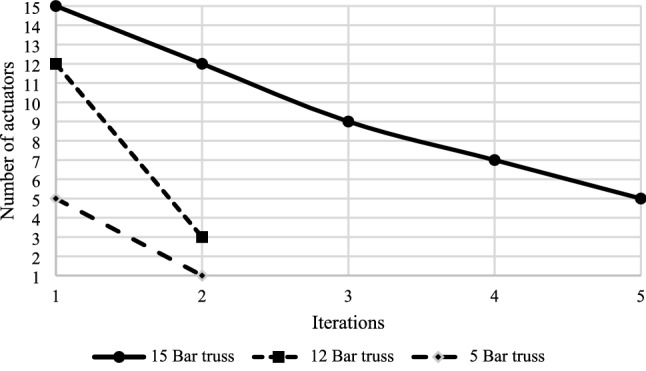
Optimization of the number of actuators.

The optimization algorithm identifies and then excludes inactive members in several iterations. The actuators’ number dropped from 5 to 1, 12 to 3, and 15 to 5 in the 5-bar, 12-bar, and 15-bar trusses, respectively (see Fig. 10). The descending slope of the 12-bar truss is more stepper than that of the 5-bar truss. This is because the total actuation for both structures is almost the same, while the participated members as actuators in the 12-bar truss in the first iteration is 12, while it is only 5 for the 5-bar truss. In the 15-bar truss, the reduction in the active members is almost linear in each iteration.

**Figure 11:**
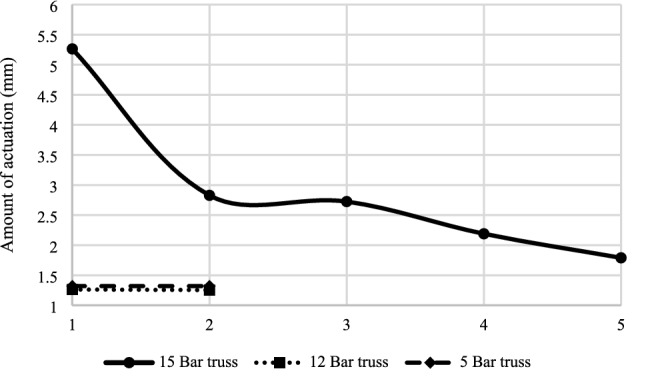
Optimization of the total amount of actuation.

Moreover, the total actuation amount declined from 1.32 mm to 1.31 mm, 1.26 mm to 1.25 mm, and 5.26 mm to 1.79 mm for the 5-bar, 12-bar, and 15-bar trusses correspondingly as presented in Fig. 11. One can see that there is a fluctuation in the total amount of actuation in five iterations for the 15-bar truss. In contrast, the amount of actuation remains unchanged in two iterations for the 5-bar and the 12-bar trusses. Furthermore, the algorithm took only two iterations to find the optimum solution for the 5-bar and 12-bar trusses, while it took five iterations for the 15-bar truss.

## Conclusion

A method based on the direct force method to control shape and stress as well as member buckling preservation was established and applied to several examples. In this study, the displacement of all joints is controlled within the desired limit; meanwhile, the tensile stress of tension members remains below the yield stress. The compressive stress of compression members stays below the critical buckling stress. Furthermore, the technique guarantees that members with S > 200 do not face compression after actuation. In addition, members' residual stress and initial crookedness were considered in critical stress calculation. Nonetheless, force method controlling equations were coupled with optimization algorithms to minimize the number of actuators and the amount of actuation. Moreover, when larger loads than designed loads are expected to be applied to a specific structure, the stress can be redistributed using the current method.

## Data Availability

The datasets used and/or analyzed during the current study available from the corresponding author on reasonable request.
